# Representations and Benchmarking of Modern Visual SLAM Systems

**DOI:** 10.3390/s20092572

**Published:** 2020-04-30

**Authors:** Yuchen Cao, Lan Hu, Laurent Kneip

**Affiliations:** 1 Shanghai Institute of Microsystem and Information Technology, Chinese Academy of Sciences, Shanghai 200050, China; caoych@shanghaitech.edu.cn (Y.C.); hulan@shanghaitech.edu.cn (L.H.); 2 School of Information Science and Technology, ShanghaiTech University, Shanghai 201210, China; 3 University of Chinese Academy of Sciences, Beijing 100049, China

**Keywords:** artificial intelligence, computer vision, SLAM, semantic scene understanding, visual localisation and mapping, spatial AI

## Abstract

Simultaneous Localisation And Mapping (SLAM) has long been recognised as a core problem to be solved within countless emerging mobile applications that require intelligent interaction or navigation in an environment. Classical solutions to the problem primarily aim at localisation and reconstruction of a geometric 3D model of the scene. More recently, the community increasingly investigates the development of Spatial Artificial Intelligence (Spatial AI), an evolutionary paradigm pursuing a simultaneous recovery of object-level composition and semantic annotations of the recovered 3D model. Several interesting approaches have already been presented, producing object-level maps with both geometric and semantic properties rather than just accurate and robust localisation performance. As such, they require much broader ground truth information for validation purposes. We discuss the structure of the representations and optimisation problems involved in Spatial AI, and propose new synthetic datasets that, for the first time, include accurate ground truth information about the scene composition as well as individual object shapes and poses. We furthermore propose evaluation metrics for all aspects of such joint geometric-semantic representations and apply them to a new semantic SLAM framework. It is our hope that the introduction of these datasets and proper evaluation metrics will be instrumental in the evaluation of current and future Spatial AI systems and as such contribute substantially to the overall research progress on this important topic.

## 1. Introduction

Research at the intersection of robotics and computer vision currently targets the development of a handful of important applications in which some form of artificial, intelligent device is employed to assist humans in the execution of everyday tasks. A few examples are given by:

- Intelligent transportation: whether talking about autonomous vehicles on open roads or Automatic Guided Vehicles (AGVs) within campuses, office buildings, or factories, transportation is a time and safety-critical problem that would benefit strongly from either partial or full automation.
- Domestic robotics: demographic changes are increasingly impacting our availability to do household management or take care of elderly people. Service robots able to execute complex tasks such as moving and manipulating arbitrary objects could provide an answer to this problem.
- Intelligence augmentation: eye-wear such as the Microsoft Hololens is already pointing at the future form of highly portable, smart devices capable of assisting humans during the execution of everyday tasks. The potential advancement over current devices such as smartphones originates from the superior spatial awareness provided by onboard Simultaneous Localisation And Mapping (SLAM) and Artificial Intelligence (AI) capabilities.

Common to these applications is their mobility and requirement of real-time operation and interaction with the real world, no matter if active or passive. The last major road-block towards the achievement of such functionality is believed to be the perception problem. More specifically, the perception problem asks for a real-time solution enabling an intelligent, embodied device to localise itself relative to its immediate environment as well as perceive the latter in the sense of generating a virtual scene representation that is useful towards the execution of a specific task.

In robotics, this problem is traditionally called Simultaneous Localisation And Mapping (SLAM). A typical realisation of a SLAM system would use either one or several exteroceptive sensors (e.g., camera, depth camera, Lidar) attached to a moving body (e.g., a human hand, a helmet, a robot) in combination with interoceptive sensors such as an Inertial Measurement Unit (IMU). The incoming sensor stream is processed in real-time to incrementally recover the relative displacement of the sensor as well as a geometric representation of the environment. While there have been many successful systems presented in the past, the generated model of the environment traditionally only covers physical boundaries in the form of low-level primitives such as a point-cloud, a mesh, or a binary occupancy grid. Such representations may be sufficient to solve the problems of path-planning and obstacle-free navigation for robots but are not yet amenable to the solution of complex tasks that require understanding the object-level composition of the environment as well as the semantic meaning of its elements.

Over the past decade, the identification of scene components (i.e., objects) and their semantic meaning has seen substantial progress through the rise of deep learning based image processing techniques. In an aim to increase the semantic, object-level understanding reflected by 3D environment representations, the SLAM community has therefore recently begun to include such algorithms for object detection and semantic segmentation in the front-end of SLAM systems and reflect the added information in an object-level partitioning and a semantic annotation of the generated 3D models. Real-time systems generating such joint geometric-semantic representations from image sequences represent an evolution of more traditional, visual SLAM into something referred to as Spatial AI (The term Spatial AI is coined by Andrew Davison in his study Future Mapping: The Computational Structure of Spatial AI Systems [1]).

In recent years, we have seen a number of exciting advancements in the development of Spatial AI systems. However, the performance of the latter is thus far mostly evaluated in qualitative terms, and we currently lack a clear definition of quantitative performance measures and easy ways to compare relevant frameworks against each other. An automated benchmark as has been introduced for traditional visual-inertial SLAM frameworks appears as an eminent gap to be filled in order to warrant research progress on Spatial AI systems. Our contributions are:

- We provide a concise review of currently existing Spatial AI systems along with an exposition of their most important characteristics.
- We discuss the structure of current and possible future environment representations and its implications on the structure of their underlying optimisation problems.
- We introduce novel synthetic datasets for Spatial AI with all required ground-truth information including camera calibration, sensor trajectories, object-level scene composition, as well as all object attributes such as poses, classes, and shapes readily available.
- We propose a set of clear evaluation metrics that will analyse all aspects of the output of a Spatial AI system, including localisation accuracy, scene label distributions, the accuracy of the geometry and positioning of all scene elements, as well as the completeness and compactness of the entire scene representation.

Our paper is organised as follows. In Section 2, we provide a review of existing Spatial AI systems. In Section 3, we introduce possible scene representations produced by a Spatial AI system that would aim at a general satisfaction of the requirements given by its applications. We also discuss how their form is reflected and used in the corresponding graphical optimisation problems. Section 4 then introduces our novel benchmark datasets including their creation, noise addition, and evaluation metrics. To conclude, Section 5 illustrates an application of the benchmark to an example Spatial AI system and discusses its performance as a representative of the current state-of-the-art.

## 2. Review of Current SLAM Systems and Their Evolution into Spatial AI

One of the first occurrences of the SLAM paradigm dates back to 1991, when Leonard and Durrant-Whyte [2] exploited a Panasonic beacon sensor to perform simultaneous localisation and mapping with a small ground vehicle robot. Meanwhile, depth measurements in robotics are generated using much more powerful laser measurement devices. A review of all SLAM solutions for all kinds of sensors would however go beyond the scope of this paper, and we focus our discussion on visual-inertial SLAM implementations that solve the problem for regular cameras that are complemented by an IMU. Our main interest lies in cameras as they deliver appearance information, a crucial ingredient for the semantic perception of environments. For a more comprehensive, general review on SLAM, the reader is kindly referred to Cadena et al. [3].

Two landmark contributions on monocular visual SLAM date back to the same year and are given by Davison’s [4] Extended Kalman Filter based Mono-SLAM algorithm and Klein and Murray’s [5] keyframe-based PTAM algorithm. The latter work in particular utilises two parallel threads to offload large-scale batch optimisation into a less time-critical background thread, a strategy that has been reused in subsequent SLAM implementations such as for example the state-of-the-art open-source framework ORB-SLAM [6]. The batch optimisation thread essentially performs bundle adjustment [7], an optimisation problem over camera poses and 3D point coordinates similar to the one solved in large-scale structure-from-motion pipelines [8,9,10]. Visual SLAM is often complemented by an Inertial Measurement Unit to robustify localisation (e.g., Qin et al. [11]).

While the most established representation for real-time SLAM systems is a 3D point cloud, the community has explored various alternatives. Delaunoy and Pollefeys [12] use a triangular mesh to directly model the surface of the environment and impose photometric consistency terms between different views. Newcombe et al. [13] introduced DTAM, a tracking and mapping approach in which dense depth information is found as a minimal cost surface through a voxel space in which each cell aggregates photometric errors between a point in a reference view and corresponding points in neighbouring frames. As demonstrated in works such as Hornung et al. [14] and Vespa et al. [15], the RGBD-SLAM community has also explored the use of binary occupancy or continuous occupancy likelihood fields. Voxel-based volumetric discretisation of space has found another application in the form of implicit, distance-field based mapping. By using RGBD sensors and the early work of Curless and Levoy [16], Newcombe et al. [17] have shown the seminal KinectFusion framework for real-time dense tracking and mapping. The technique has inspired many follow-up contributions, including recent ones that bridge the gap to Euclidean distance fields [18] and fuse multiple subvolumes for global consistency [19]. Further popular alternatives for structure representation are given by lines [20,21] or surfels, the latter one being utilised in Whelan et al.’s ElasticFusion algorithm [22].

A commonality to all the above-mentioned algorithms is that they employ either explicit or implicit low-level primitives for representing the environment (i.e., points, lines, triangles, or grid cells). Each element is constrained directly by the measurements, and resilience with respect to noise and missing data may be increased by enforcing local smoothness constraints. However, the mentioned approaches do not impose less localised constraints given by semantic or other higher-level information about certain segments of the environment. As a simple example, consider a situation in which we would know that an entire set of 3D points lies on a plane. We would prefer to model this part of the environment using a single instance of plane parameters rather than many 3D points. However, such strategy assumes the existence of a front-end measurement segmentation module able to detect meaningful parts of the environment (e.g., planes, objects of a certain class), establish correspondences between such measurement and scene segments, or even understand certain geometric properties about them (e.g., 3D object pose). Examples of such front-end modules are given by the Yolo9000 object detection framework [23], the Mask-RCNN framework for instance-level segmentation [24], Chen et al.’s hybrid task cascade framework for instance-level segmentation [25], or PointRCNN for 3D object pose estimation in 3D point clouds [26], to name just a few.

A system that is able to then incorporate higher-level knowledge such as learned semantic priors into the 3D representation of a real-time mobile visual perception system is defined in Davison’s studies FutureMapping 1 & 2 [1,27] as a Spatial AI system. In other words, a Spatial AI system is characterised by generating a joint geometric-semantic 3D understanding about an environment. In its most basic form, the extracted semantic front-end information is simply used to perform a segmentation and labelling of the 3D representation of the environment. Choudhary et al. [28] and Gálvez-López et al. [29] are among the first to propose a real-time SLAM system that actively discovers and models objects in the environment. Later, similar object-detector-based systems have been introduced by Sünderhauf et al. [30] and Nakajima and Saito [31]. Stückler et al. [32], McCormac et al. [33,34] and Pham et al. [35] in parallel investigated the semantic 3D segmentation and labelling of dense representations. While initially only at the level of object-classes, Grinvald et al. [36] and Rosinol et al. [37] have most recently investigated object-instance level 3D segmentations of the environment.

As also outlined in Davison’s studies [1,27], there are further desired properties of a Spatial AI system, which are memory efficiency and the imposition of higher-level prior knowledge into the geometric representation of the environment. While the above-mentioned systems already maintain a graphical model describing the object-level structure of the scene as well as the observabilities of objects in frames, complete surface representations still ask for dense, low-level representations to model the geometry of each individual part of the environment [33,34]. The most straightforward way to reduce the dimensionality and employ higher-level priors is given by omitting shape optimisation altogether and employing complete object geometries such as CAD models as known priors. This strategy is pursued by the seminal work SLAM++ by Salas-Moreno et al. [38], which employs a graphical model that simply parametrises camera and object poses. A more flexible approach was later proposed by Ulusoy et al. [39], where the assumption of a known CAD model is relaxed to a fixed set of possible 3D shapes. Their work focuses on photometric multiview stereo, and the optimisation of the pose of each object is complemented by a probabilistic, discrete selection of the best element within the shape set. Gunther et al. [40] propose a similar framework for indoor modelling with a given set of CAD models of pieces of furniture. Another interesting way of including semantic knowledge is proposed by Häne et al. [41], who use the semantic information to adapt the smoothness constraint between neighbouring voxels in a dense representation.

One obvious disadvantage of frameworks such as SLAM++ [38] is that they are limited to a small set of possible 3D shapes which are given upfront. In other words, they do not employ a generic class-specific model that would permit the continuous optimisation of a given object shape. As introduced in [42], low-dimensional shape representations can be obtained using one of a few approaches of manifold learning (e.g., PCA, kernel-PCA, Isomap, LLE, auto-encoder). However, unsupervised learning and the resulting optimisability of such representations are far from trivial, which is why the first lower-dimensional models employed in the literature are explicit. Güney and Geiger [43] and Chhaya et al. [44] employ class-specific, optimisable meshes or wireframe structures, respectively. Hosseinzadeh et al. [45] and Gay et al. [46] employ quadrics as shape primitives to approximate the space occupied by certain objects.

Approaches that finally achieve dense, optimisable environment representations by low-dimensional graphical models and implicit, class-specific shape representations are given by Dame et al. [47] and Engelmann et al. [48], who both rely on PCA to learn a manifold for an admittedly simple class of shapes: cars. Alismail et al. [49] and Zhu and Lucey [50] later on propose more advanced frameworks that rely on deep neural networks to generate point clouds or occupancy grids from the latent low-dimensional representation. Hu et al. [51] finally extend this approach to a complete SLAM framework that optimises a larger scale graph over many frames and multiple complex-shaped objects of different classes (e.g., chairs, tables) as well as latent shape representations for each object instance in parallel. Note that low-dimensional latent representations for modelling 3D geometry have also been utilised by Bloesch et al. [52] and Zhi et al. [53]. More specifically, they rely on photometric consistency to optimise codes in each keyframe that generate depth maps using a deconvolutional architecture. The representation however seems suboptimal as it does not respect object-level partitioning and furthermore leads to redundancy (i.e., one depth map in each keyframe but a potentially large overlap between neighbouring keyframes).

Spatial AI systems that aim at a real-time understanding of both the geometry and the semantics of an environment generally contrast with more time consuming offline approaches that start from known camera poses and known low-level geometry only to infer an object-level representation of the scene. For example, Gupta et al. [54] and Li et al. [55] make use of a large-scale database of 3D CAD models to estimate object poses and geometries and replace parts of the 3D geometry. Chen et al. [56] further rely on contextual relationships learned from the 3D database to constrain the reconstruction. More recently, Huang et al. [57] propose holistic scene grammars to infer scene layouts from single images based on analysis by synthesis. The nondifferentiable optimisation space is traversed using Markov Chain Monte Carlo (MCMC). In contrast, Grabner et al. [58] propose a discriminative approach to predict an object model and its pose. Although interesting and related, the listed scene layout estimation frameworks are not designed for high efficiency and rely on large databases of object shapes rather than compact, optimisable shape representations.

## 3. What Representations Are Pursued by Spatial AI?

**Figure 1:**
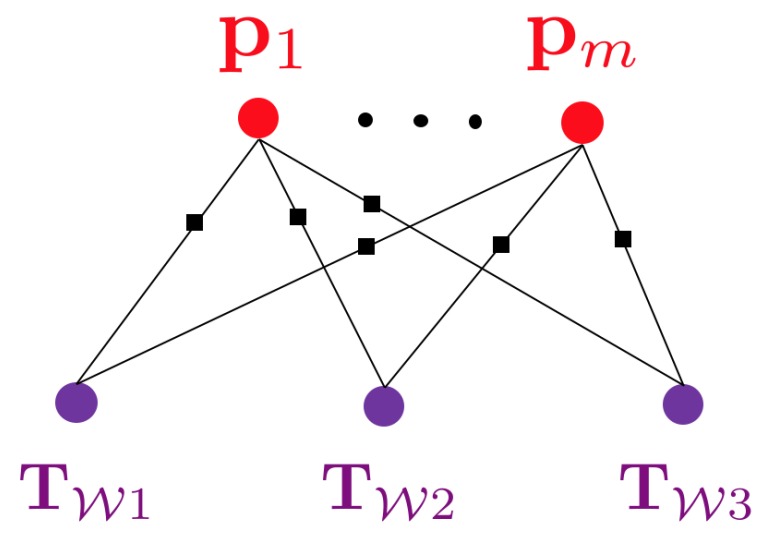
Factor graph of a classical visual Simultaneous Localisation And Mapping (SLAM) problem in which multiple views observe multiple 3D landmarks.

Classical solutions to camera-based SLAM rely on least-squares optimisation objectives that consist of minimising many localised photometric or geometric residuals over many views. They depend on an explicitly parametrised, high-dimensional, and low-level representation of the environment. Examples of the latter are given by geometric primitives such as 3D point clouds, sets of lines parametrised in 3D, voxel-based occupancy maps or distance fields, or even surface representations in the form of a triangular mesh. The resulting optimisation problems often have a graphical representation in which nodes represent optimisation variables (e.g., camera poses, 3D landmark coordinates) and edges represent correspondences between 3D points and frame observations and thus locations in which the relative positioning between the related variables can be cross-validated against the actual measurements. As introduced by Dellaert and Kaess [59], such graphical models are often referred to as factor graphs. A classical example is illustrated in Figure 1.

While possibly giving us accurate information about the physical boundaries of an environment, such representations generally do not reveal anything about the composition or semantics of a scene. As the entire modelling process purely relies on the online measurements, the robustness of classical methods is furthermore easily compromised by unmodelled effects such as occlusions or violations of assumptions made on surface reflectance properties (e.g., in computer vision, we often assume Lambertian surfaces such that the coordinates of a light source or the appearance changes under varying camera inclinations do not have to be taken into account). In the following, we will discuss a series of forward-looking environment representations, their use within SLAM algorithms, as well as expected resulting benefits.

### 3.1. Hybrid Graphical Models

Our first step towards the proposal of a new environment representation employed in future spatial AI algorithms consists of introducing naturally occurring segmentations. For example, indoor man-made environments are naturally composed of a background structure filled with objects that have certain geometric and semantic properties. It makes sense to adopt this partitioning for the internal environment representation used within a SLAM algorithm for the following reasons:

- It gives us more flexibility in choosing different but more appropriate parametrisations for the geometry of each segment.
- It gives us a reasonable partitioning according to human standards enabling us to assign a class or—more generally—semantic attributes to each segment.

**Figure 2:**
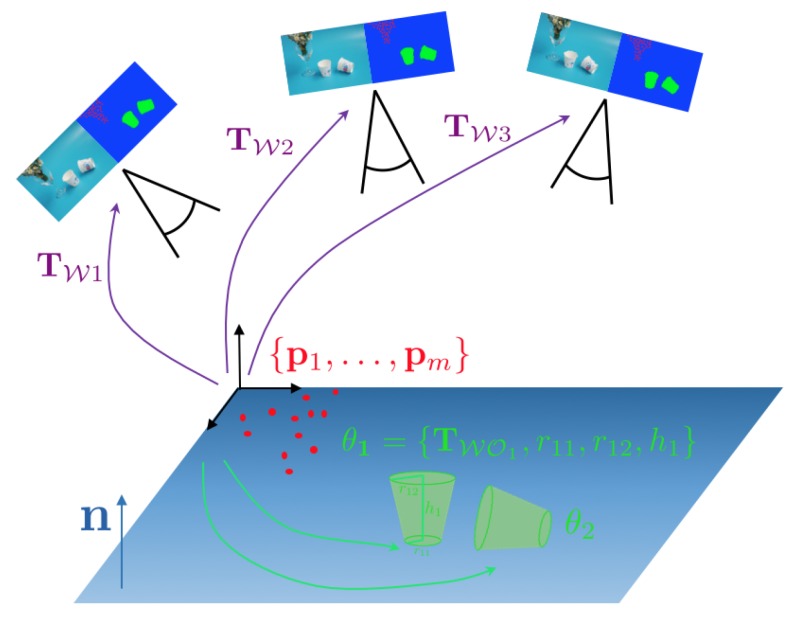
Visual SLAM scenario in which a camera observes a simple Desktop scene. The environment is represented as a hybrid composition of objects and primitive geometric elements (e.g., points and planes).

Figure 2 indicates a desktop-scale example in which a camera moves in front of a table on which we have two paper cups and a plant. By using object detections or semantic segmentation results predicted in each view, we would start by defining the representation of the environment as the following set of variables:

- Two instances of a thin conical object with location and shape parameters. Due to rotational symmetry, the pose $T_{\mathrm{WO}}$ would have 5 Degree of Freedom (DoF), and the number of shape parameters would be 3 (bottom radius $r_{1}$, top radius $r_{2}$, and height of the object *h*).
- One set of 3D points $\left\{p_{1},\ldots ,p_{m}\right\}$ to represent the plant. The cardinality of the set is, for the example, given by the number of sparse, multiview feature correspondences between the images.
- A plane vector n to represent the background structure.

**Figure 3:**
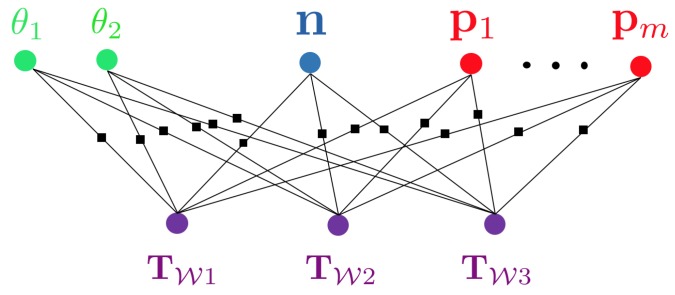
Factor graph corresponding to the example shown in Figure 2. Each factor in the graph (indicated by a black box) represents a residual error from an incidence relationship between measurements and a combination of a camera pose and some object or background geometry related parameters.

Using an algorithm to establish correspondences between views, we can finally define the graphical structure of this optimisation problem. Figure 3 shows the resulting factor graph in which each factor represents a residual error of an incidence relationship between measurements and a combination of camera pose, background structure parameters, sparse 3D point location, or object pose and shape. Owing to the fact that it involves objects of different classes or levels of abstraction (i.e., points, planes, shapes), we denote such a type of problem a hybrid graph optimisation problem.

### 3.2. Hierarchical Hybrid Graphical Models

**Figure 4:**
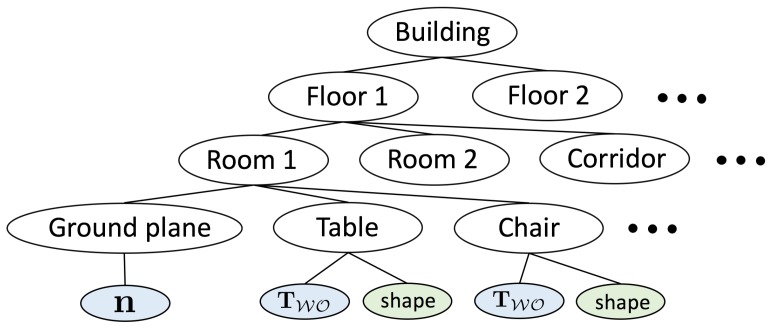
Example hierarchical, tree-based structure of man-made indoor environments.

One further aspect that is still missing in the envisaged representations is the naturally occurring hierarchical nature of man-made structures. We again start from the example of indoor man-made environments, which have a hierarchical, multiscale subdivision into buildings, floors, and rooms (cf. Figure 4). Establishing and reconstructing such hierarchies within visual SLAM algorithms may lead to the following strong benefits:

- A natural subdivision of the graph into multiple subgraphs, which is a requirement in larger-scale applications where the complete map can no longer be loaded into memory. The hierarchical, tree-based structure of the map permits loading submaps of different scales (e.g., entire floors or just single rooms).
- An agreement between the tree structure of the map and the natural segmentations and hierarchical subdivisions used by humans, which simplifies man-machine interaction.
- Parent structures appearing as separate nodes in the optimisation graph and enabling the construction of higher-order residual errors.

**Figure 5:**
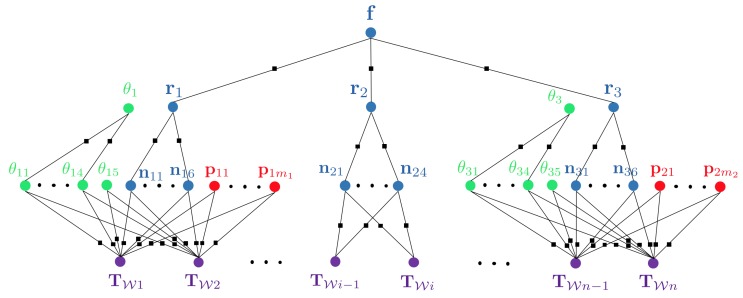
Factor graph for a visual SLAM problem in which a robot moves from one room through a corridor into another room. Both rooms contain 4 chairs of similar shape and one table. The hierarchical structure of the environment is exploited for the imposition of higher-order constraints. The bottom level of the environment representation consists of object parametrisations ($\theta_{11},\ldots ,\theta_{14}$ represent chairs in room one, $\theta_{31},\ldots ,\theta_{34}$ chairs in room 3, and $\theta_{15}$ and $\theta_{35}$ tables in room 1 and 3, respectively), background planes $n_{xy}$ (6 for room 1 and room 3, and 4 for room 2, which is the corridor), and point clouds for the representation of unstructured segments of the environment. At the centre level, we have variables $r_{x}$ which parametrise the different rooms in the environment (e.g., parameters of a rectangular footprint and a room height). The factors between the bottom and the centre layer enforce the consistency of the room parameters and the individual planes, and thus implicitly enforce higher-order constraints such as orthogonality of planes. We furthermore have one average parametrisation $\theta_{x}$ used to enforce the similarity of all objects of similar shape within each room. Factors between the centre and the top layer finally constrain the individual room parameters to be consistent with some floor parameters f (e.g., the vertical coordinate of the floor may be enforced to be the same in each room).

To illustrate the latter argument, we again picture a concrete scenario in which a floor cleaning robot navigates in an office building. Let us imagine that—after traversing the first room—it passes through a corridor and enters another room to continue its task. Each room has a rectangular footprint with vertical walls and is furnished with one table and four chairs, the chair shapes being the same within each room. The resulting graphical model that exploits the hierarchical structure of the environment is indicated in Figure 5.

### 3.3. Expected Advantages of the Proposed Environment Representations

The envisaged environment representations aim at a radical change in how we represent and optimise environments in visual SLAM. Inspired by the recent success of deep learning in computer vision, the hierarchical object-level representations of the environment leverage artificial intelligence to identify the segmentation and composition of an environment. However, deep learning may not only be employed for solving front-end tasks such as the segmentation of images and the initialisation of a graphical model as outlined in Section 3.1. Auto-encoder architectures have successfully been employed to perform dimensionality reduction on high-dimensional object shape representations, thus leading to low-dimensional embedding spaces to parametrise and constrain the shape of an object of a certain class [60].

The expected benefits of the generated representations therefore are:

- Compact map representations: whether talking about a planar surface representing a wall or a more complex object shape, the object-level partitioning permits the choice of class-specific implicit low-dimensional representations. While choosing a simple three-vector to parametrise a plane, more complex objects could be modelled using the above-introduced, artificial intelligence based low-dimensional shape representations. The compact representations have obvious benefits in terms of memory efficiency, a critical concern in the further development of next-generation Spatial AI systems.
- Low-dimensional representations: by employing class-specific, we implicitly force optimised shapes to satisfy available priors about their geometry. In contrast to current approaches which use high-dimensional, semantically unaware representations that only employ low-level priors such as local smoothness, the (possibly learned) higher-level priors we may employ for certain objects of known classes are much more powerful and implicitly impose the correct amount of smoothness for each part of the object. It is conceivable that such techniques will have a much better ability to deal with measurement disturbances such as missing data or highly reflective surfaces.
- Current low-level representations enable the automated solution of low-level problems such as the efficient, collision-free point-to-point navigation in an environment but do not yet enable the execution of more complex instructions that would require an understanding of semantics and composition. Hierarchical hybrid graphical models would give machines a much more useful understanding of man-made environments and notably one that is presumably much closer to our own, human reasoning.

## 4. A New Benchmark

We now proceed to the introduction of our new benchmark for Spatial AI systems. After a review of existing datasets in the literature, we present our new synthetic datasets along with their main advantages, creation, and evaluation metrics.

### 4.1. Review of Existing Datasets and Benchmarks

Numerous visual-sensor-based datasets have been publicly released for different purposes and made great contributions in the SLAM and the deep learning communities. Within the SLAM community, the TUM-RGBD [61] and the KITTI [62] datasets are noteworthy examples that use custom camera arrangements for specific scenarios such as indoor SLAM or self-driving cars. The datasets are fully calibrated, come with ground-truth, and even provide full online benchmarking (i.e., they provide additional examples for which ground truth is disclosed and for which a server can automatically evaluate submitted results). Within the learning community, a notable example of a dataset for semantic image labelling is given by Microsoft’s Coco Dataset [63], the result of an extreme manpower and time effort to mask semantic ground truth labels of 1.5 million images over 80 object categories. Similar examples are given by NYU’s Depth Dataset [64], which includes depth images that can be used to complement the network input. The ADE20K dataset [65,66] contributes further with fully semantically annotated datasets with detailed attributes such as cropped and occluded situations, yet it does not provide 3D information. Another interesting work combining 2D and 3D information is given by the Sun3D dataset [67], which uses weak manual labelling of semantic objects in a subset of frames and utilises structure from motion to propagate the semantic 2D and 3D labels into neighbouring frames. The method, however, depends on robust propagation and rich loop closures to ensure the accuracy of the labelling.

Predictions from 2D images (even if complemented by depth information) easily suffer from the challenge given by occlusions in the data, which is why the current research trend shifts the focus from an evaluation over 3D instead of only 2D data. Novel datasets containing ground truth information about the contained 3D objects along with their semantics, poses, and shapes are therefore in great demand. A solid starting point in this direction is given by Stanford’s 3D Scene Datasets [55] and ScanNet [68]. In order to gain object-level ground truth for their datasets, they retrieve similar 3D models from the online database ShapeNet [69], which are subsequently aligned with the measurements. While this technique works reasonably well, it suffers from two problems. First, the alignment is influenced by the quality of the measurements, which poses a challenge in the presence of noise, outliers, and partial measurements. Second, the models retrieved from ShapeNet may not have the exact same 3D shape as the real-world models, thus leading to discrepancies in the ground truth object shape as well. In summary—although these datasets are useful to develop novel methods and perform qualitative comparisons—they miss accurate ground truth data that could be used to perform quantitative comparisons for both object poses and shapes. A partial remedy to this is given by the synthesised realistic datasets presented by Alhaija et al. [62], who perform augmented reality to insert virtual models synthesised by a neural network into the original KITTI image sequences. While this gives us exact ground truth knowledge about the 3D pose and shape of the contained objects, the approach is still limited in that objects are always generated while fully visible in the foreground and never have consistent illumination. In other words, the approach does not realise complex mixed reality scenarios in which an arbitrary number of objects could be inserted at arbitrary places in the real world, and all resulting occlusions would be perfectly modelled.

**Figure 6:**
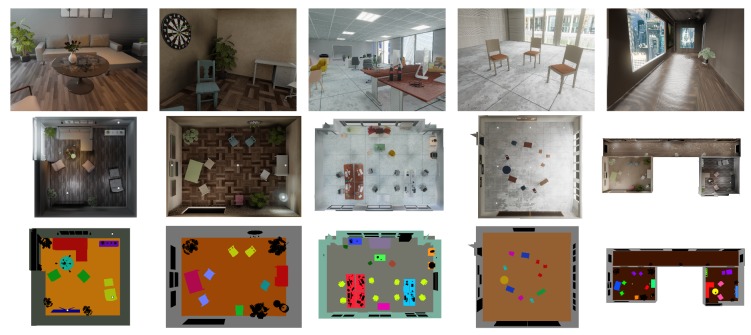
An overview of five provided datasets with RGB images from the sequence at the top, a top view of the scene in the middle, and a top view of the semantic maps at the bottom.

Our novel benchmark datasets are motivated by this circumstance and inspired by the ICL-NUIM datasets [70], which introduce a method to synthesise photo-realistic image sequences from virtual environments with adequate light tuning and noise addition. Our datasets provide similar synthetic and photo-realistic image sequences of complex indoor scenarios, along with stereo RGB images, RGB-D depth images, and 6-axis IMU readings. We furthermore complement the commonly available ground-truth data of camera trajectories by semantic instance-level labels and full information about the scene composition along with the ground truth 3D models and poses of semantic objects. Figure 6 gives an overview of our datasets. The datasets are available on our website http://mpl.sist.shanghaitech.edu.cn/SSSBenchmark/SSS@MPL.html.

### 4.2. Dataset Creation

In recent times, the rapid development of Graphics Processing Unit (GPU) performances and computer graphics algorithms eases the efficient generation of highly realistic virtual datasets. The main advantages of synthetic datasets are:

- Rendering engines model the imaging process according to some preset parameters and with a unified computer clock for each virtual sensor. This effectively saves the effort for intrinsic and extrinsic calibration of the multisensor system, including the tedious time stamp alignment between RGB, depth, and semantic images as well as the IMU readings.
- The convenience given by the software-based adjustment and replacement of objects and cameras vastly increases the efficiency of the entire dataset generation process.
- The diversity of the freely available, virtual 3D object models including the variability of their texture maps enables the automatic generation of a large number of highly variable datasets, which improves the generality of the subsequently developed Spatial AI, SLAM, and deep learning algorithms. If designed well, the dataset properties including the virtual multisensor setup, the sensor trajectory, the scene layout, and the objects’ composition, arrangement, and appearance can be steered at the hand of a simple input script.

We use the Eevee renderer in Blender 2.8 to generate our datasets on a workstation with GTX 1050 Ti and i7-7700 CPU. We provide a complete and easy-to-use framework for the generation of novel datasets and five pregenerated datasets with certain characteristics for the development of future indoor Spatial AI systems. Table 1 summarises the provided datasets with scale, diversity and classes of objects, as well as occlusion and camera motion properties.

#### 4.2.1. RGB Map

To get photo-realistic RGB data, we set up proper irradiation of sun and lamps, map objects with 4K high-quality textures and HDRI from https://www.CC0textures.com/ and https://www.poliigon.com, and adjust reflection and transparency to guarantee the complexity and authenticity. Our primary output is stereo RGB sequences for research on stereo SLAM systems. Both cameras have a default framerate of 30 fps, a baseline of 0.12 m, a CCD sensor size of 35 mm, a configurable horizontal FOV of 72.7 degrees (24 mm focal length), an F-stop of F22, and SVGA resolution (i.e., 1024 × 768). The intrinsic parameters are summarised as

(1) $$K=\left[\begin{array}{ccc} 702.127 & 0 & 512.0\\ 0 & 702.127 & 384.0\\ 0 & 0 & 1 \end{array}\right].$$

We furthermore add noise to the rendered datasets to simulate the behaviour of real-world cameras. Normal DSLR cameras contain three types of noise: photon shot noise (PSN), read noise (RN), and PRNU (Pixel Response Non-Uniformity) noise. Their relation with the raw image pixel brightness $B_{raw}$ is given by

(2) $$B_{raw}=\left(\mathrm{PSN}+\mathrm{PRNU}\right)\cdot \mathrm{EL}+\mathrm{RN}-\mu_{RN},$$

EL represents the amount of electrons assembled in the optical sensor during a period of exposure time. $\mu_{RN}$ is given by the camera’s denoising process encountering the read noise, hence the latter can be ignored. Photon shot noise follows a Poisson distribution and in most cases, it is close to a normal distribution. PRNU varies for different photons and can be regarded as a normal distribution. According to [71], the Camera Response Function (CRF) transfers the linear high dynamic range $B_{raw}$ to the nonlinear low dynamic range B at the output. We refer to [70] and reformulate (2) into

(3) $$B=crf(\frac{\sum_{i=1}^{N}B_{raw}}{N}+N\left(0,\sigma_{e}\sqrt{\frac{\sum_{i=1}^{N}B_{raw}}{N}}\right)+N\left(0,\sigma_{p}\right)),$$

where $B_{raw}$ contains the exposure time Δt, and *N* can be calculated from N=fps·Δt. We set $\sigma_{e}=0.05$ and $\sigma_{p}=0.02$ for our case. However, there is no setting for the exposure time Δt in Blender. We propose a work-around to calculate the exposure time *S* from the equation

(4) $$EV=ISO_{100}+log_{2}\frac{ISO}{100}-log_{2}\frac{A^{2}}{S}.$$

**Figure 7:**
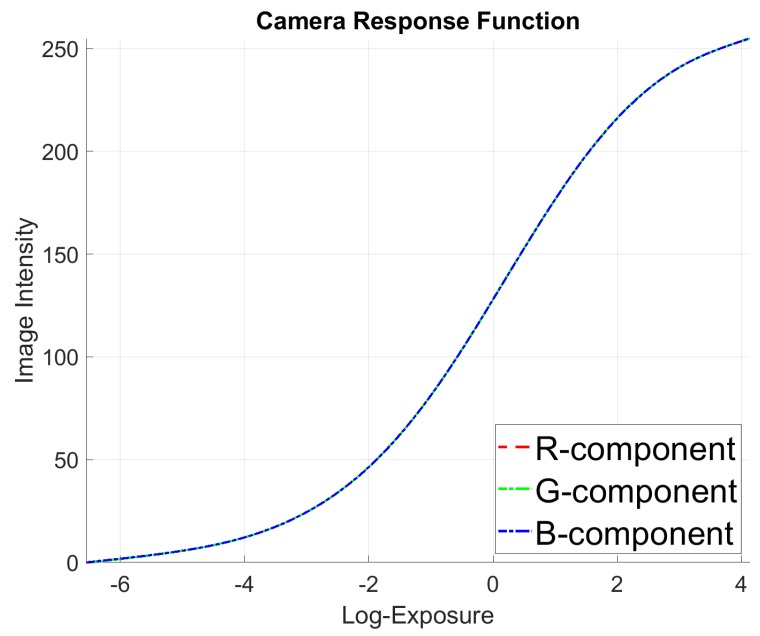
The Camera Response Function (CRF) curve calculated with images of different exposure time in the same static scene.

We set $ISO_{100}=9.67$ from the ISO 100 chart and the aperture to A=F11 for indoor scenarios. By defining the virtual ISO as 640, the exposure time *S* can be calculated by setting the EV value in Blender. We set EV to integer values within the range [−9,−8,...,4,5] (low-exposure images are calculated due to the fact that dark pixels are more sensitive in terms of their CRF) to render images of different exposure in a static scene. Through experimentation, we finally choose the best-fitting CRF conversion curve (cf. Figure 7). We furthermore test the CRF in different scenes to revert RGB to linear space and compare with EXR data exported from Blender and list the error in Table 2.

**Figure 8:**
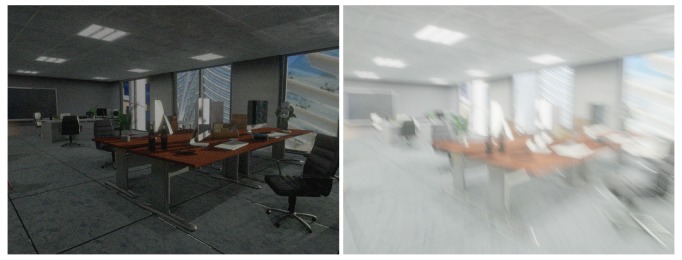
The left image is generated with fps=30 Hz and EV=−3 (exposure time: 0.0029 s). The result is a noisy, low-intensity image; The right imageis generated with fps=120 Hz and EV=1 (exposure time 0.0464 s). As a result, we obtain significant motion blur and higher intensity.

To add noise to the RGB images, we set ISO and *A* static, and tune the frames-per-second and exposure time to get different types of noisy and blurred RGB images (cf. Figure 8).

#### 4.2.2. Depth Map

To support research on RGBD-SLAM systems, we furthermore synthesise depth images from a depth camera that coincides with the left view of the stereo camera. We scale down linear raw depth and export images without colour space conversion. This comes at the cost of a slight loss of depth information beyond 20 m as we use only the 16-bit grayscale format to balance visualisation and accuracy. A single bit therefore represents 0.305 mm of real distance.

Realistic depth data captured from a real-world camera always contains noise. We break down depth noise into three parts: lateral noise, disparity noise, and depth axis noise.

For lateral noise, [72] provides a method to generate Kinect-like depth. They first compute the angle between the camera ray and the surface normal corresponding to each 3D point from depth image, then use a threshold of angle to constrain the valid depth value. In our case, we adjust the threshold that any angle larger than 82 degrees is taken as an invalid pixel as the reflection of the infrared light is considered to be out of the receiver’s range, thus causing a collapse of the measured depth. We furthermore implement the method from [72] to extract canny edges in the depth image and disturb the depth value along the gradient direction. The disturbance is sampled from a normal distribution with mean 1 and standard deviation 1 (in unit pixels), to generate intersected edges between the background and the objects.

Most depth sensors consist of an emitter and a receiver, accounting for baseline noise. On the receiver side, the reflection of an infrared ray from the background can be occluded by objects. Furthermore, empty pixels are caused by missing receptions of reflected rays. We define another virtual depth camera with a horizontal baseline of 0.075 m to the left and warp the depth map using

(5) $$Z_{right}\left(x+\Delta x,y\right)=min\left(Z_{left}\left(x,y\right),Z_{left}\left(x_{i},y\right)\right),$$

(6) $$\Delta x=\frac{baseline\cdot f_{p}}{Z}.$$

Given that the sensors have an ideal shift along the *x*-axis, the difference along the *y*-axis can be ignored. We use bilinear interpolation to find the nearest pixel coordinates in the right image and record the smallest $Z_{left}$ that project onto that pixel. The nonvisited pixels are labelled as invalid. A gradient filter along the *x*-axis is applied to smooth out wrongly labelled invalid pixels from the coordinate approximation.

For the depth axis noise, we refer to the method in [70], where the background is given high noise and closer objects are given low noise to guarantee high SNR. We therefore adjust the disparity equation as in

(7) $$\frac{\alpha \cdot baseline\cdot f_{p}}{Z_{new}}=\frac{\alpha \cdot baseline\cdot f_{p}}{Z_{old}\left(x+nx,y+ny\right)}+N\left(0,\sigma_{d}^{2}\right)+\delta_{s}.$$

**Figure 9:**
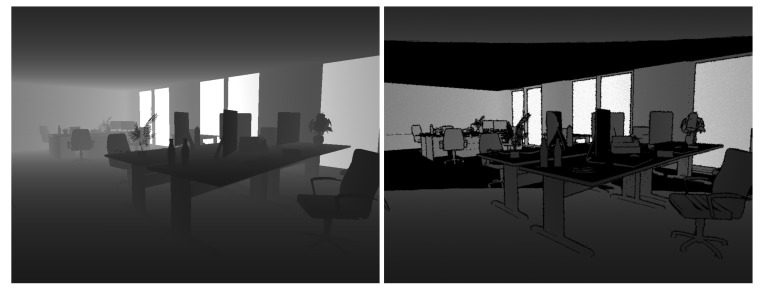
Comparison of a rendered depth image from Blender and a noise-processed realistic depth image. The noisy depth image includes disturbances along the edge, depth collapse, and reserved SNR.

In our case, (nx,ny) is normally distributed with 0 mean and a standard deviation of 0.5 ($\sigma_{d}=0.5$), and $\delta_{s}=0.2$. baseline=0.075 is a virtual horizontal baseline between emitter and receiver, $f_{p}$ is the focal length in pixels, and α is a parameter that tunes the depth noise distance. Figure 9 shows an example of a noisy depth map.

#### 4.2.3. Segmentation Map

**Figure 10:**
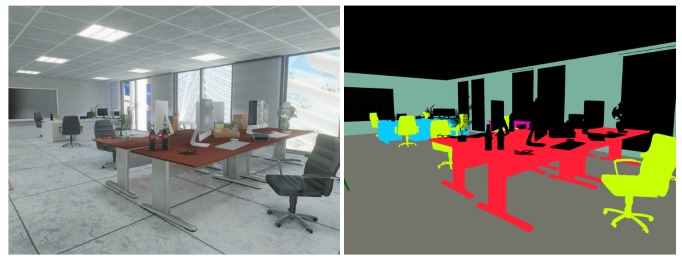
Semantic segmentation image (right) corresponding to an RGB image (left). The black colour is a random object, and instances with the same class label are marked in the same RGB color.

As the last ingredient, we add high-quality semantic segmentations to assist with the tasks of finding, implementing, and tuning front-end modules for newly designed Spatial AI systems. The ground truth semantic segmentations are again generated in camera views that coincide with the left stereo view. They are generated by replacing the texture maps with uniform RGB colours for each object instance. The colour is notably fixed by the class label. We furthermore eliminate all light sources to make sure that no shading on the objects appears. We use 16-bit PNG format to limit each object’s interior colour variations within one bit. An example set of images for a single time instant in one of our datasets is illustrated in Figure 10.

#### 4.2.4. Camera Trajectories

Next, we discuss the definition of the camera trajectories. Realistic trajectories could be obtained by running a SLAM algorithm over real image streams captured by a handheld or a robot embedded camera and using them for the virtual camera in the synthetic scene. However, the sparse and uneven timestamps between keyframes gained from a SLAM tracker do not serve well for the generation of IMU data and limit the flexibility in customising the framerate or the actual trajectory of the virtual camera. We therefore synthesise smooth trajectories emulating handheld camera motion for all datasets. The camera in Blender follows a convention different from the one used in computer vision (i.e., *z*-axis points forward, *x*-axis points right, and the *y*-axis points downward), which is why Blender transformation matrices $T_{blender}$ need to be rectified following the equation

(8) $$T_{groundTruth}=T_{blender}\cdot \left[\begin{array}{ccc} 1 & 0 & 0\\ 0 & -1 & 0\\ 0 & 0 & -1 \end{array}\right].$$

We use Bezier curves to generate smooth trajectories for cameras to follow. The control points of the poly-Bezier curves also let angular velocity vary smoothly. Keyframes are distributed evenly along the curves, and linear velocity can be controlled by scaling the keyframe timestamps. In order to simulate the shakiness of handheld cameras, we furthermore add different F-curve perturbations along each axis of rotation and translation. By tuning both the phase and frequency of these perturbations, the camera may be configured to exert motion similar to a real camera.

#### 4.2.5. IMU Data

To obtain reliable IMU data, we increase the timestamp resolution by a factor 10 (i.e., 300 Hz for a 30 fps camera). Simulated IMU accelerations and angular velocities are finally obtained by numerical differentiation. Accelerations are derived using

(9) $$a=\frac{p_{t+1}+p_{t-1}-2p_{t}}{\Delta t^{2}}$$

The effect of gravity is subsequently accounted for by subtracting 9.8 m/s${}^{2}$ along the vertical direction. To conclude, the readings are transferred from the global to the local camera frame (i.e., the extrinsic transformations between the camera and the IMU are set to an identity transformation). Next, we use Rodrigues’ formula to calculate the angular velocity ω

(10) $$W=\frac{1}{2(t_{1}-t_{0})}\frac{\theta }{sin\theta }\left(A-A^{T}\right),$$

(11) $$\omega =[-W_{12},W_{02},-W_{01}],$$

where A represents the relative rotation from frame $R_{1}$ to frame $R_{0}$, and θ the angle of rotation:

(12) $$A=R_{1}R_{0}^{T},\theta =cos^{-1}\left(\frac{tr(A)-1}{2}\right)$$

To conclude, the angular velocities are again rotated into the respective camera frame.

The ground truth data of the trajectories are recorded line-by-line in a separate file, where each line contains the pose variables in the order (timestamp,x,y,z,qw,qx,qy,qz).

Furthermore, for IMU data is in the order (timestamp,x,y,z,radx,rady,radz). The unit for translations is in meters, and the time unit for timestamps is $\frac{1}{30}$ s.

#### 4.2.6. Semantic Object Data

**Figure 11:**
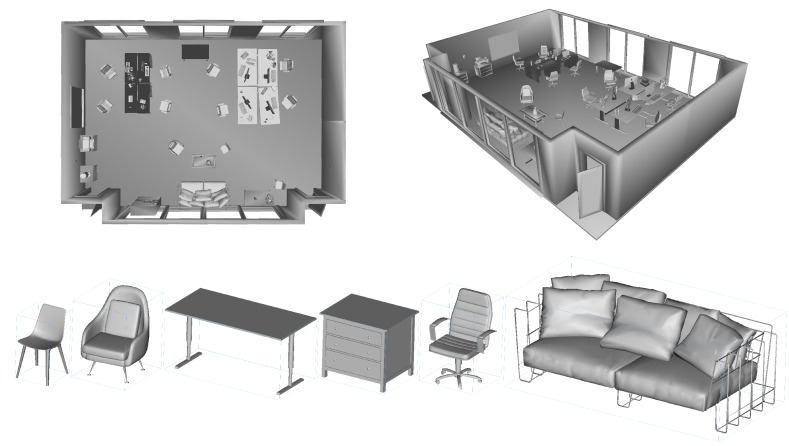
The geometry of rm3 with the placement of semantic objects and room environment as an illustration of the composition of our dataset. The image below shows calibrated semantic objects in their initial canonical poses.

Next, we discuss the definition of the environment. An example with individual objects and their arrangement is illustrated in Figure 11 for dataset rm3. The contained object models range from simple clean-cut geometries to complex folded cloth meshes. The models are either generated by ourselves or downloaded from the 3D model website TurboSquid (https://www.turbosquid.com/). We reduce the face and vertex resolution of each object to minimise physical size without damaging the quality of the shapes. The transformation matrix (i.e., pose) of each semantic object is stored along with its class label in an individual file. We also provide one .obj model for each added type of object and the empty room.

### 4.3. Dataset Toolset

**Figure 12:**
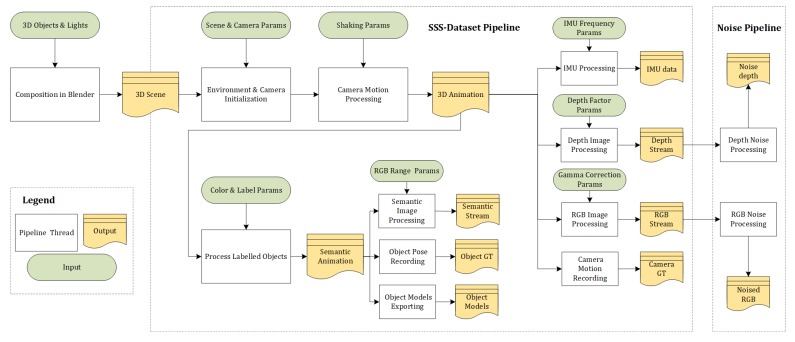
Pipeline for generating datasets. The green boxes represent parameters that can be tuned for customised dataset generation, the white boxes are core codes to generate results in yellow boxes, including RGB, depth and semantic maps, camera poses, object poses, object 3D models, IMU data, and depth and RGB noise.

We open-source our scripts for the generation and noise addition of datasets under the link https://github.com/CaoYuchen/SSS-dataset. The purpose is to supersede complex manual operations in Blender and merge our data processing methods with the Blender API and also provide realistic sensor noise processing for RGB and depth data. The scripts also allow users to simply tune input parameters for the generation of customised datasets. We expect it can be furthermore used in-the-loop inside a (non-real-time) Spatial AI system that operates by the analysis-by-synthesis paradigm. Figure 12 explains the entire pipeline of our dataset generation scripts.

Table 3 demonstrates the average durations in unit seconds to generate a single image. The last row demonstrates the RGB and depth noise processing time for each image. The time measurements are recorded on a Windows 10 system with an eight-core i7-7700 CPU and a GTX 1050Ti.

### 4.4. Evaluation Metrics

The proposed metrics for Spatial AI systems evaluate all aspects of the Hybrid Graphical Representations introduced in Section 3.1. This includes the quality of the poses of the virtual sensor setup for every image in the sequence, the correctness of a label distribution over the identified objects in the environment, and the correctness of the class, pose, and shape for every correctly identified object in the environment. In the following, we will introduce all evaluation aspects along with their metrics.

#### 4.4.1. Evaluation of Sensor Poses

In order to evaluate the localisation accuracy of a Spatial AI system, we reuse two standard measures which have also been used by the TUM-RGBD benchmarking [61] frameworks:

- Average Trajectory Error (ATE): The Average Trajectory Error directly measures the differences between corresponding points of the ground truth and the calculated trajectories. The evaluation is preceded by a spatial alignment of these two trajectories using a Procrustes alignment step. The latter identifies a Euclidean or similarity transformation that aligns the two trajectories as good as possible under an L2-error criterion. The residual errors are due to drift or gross errors in the sensor motion estimation, which the ATE characterises by either the mean, median, or standard deviation of the distance between the estimated and the ground truth position:
  (13) $$\mathrm{ATE}=\left\{\begin{array}{c} \mathrm{mean}\\ \mathrm{median}\\ \mathrm{std}-\mathrm{dev} \end{array}\right\}\left(\begin{array}{c} \|t_{\mathrm{GT}}\left(t_{0}\right)-Qt_{\mathrm{est}}\left(t_{0}\right)+\mu \|\\ \cdots \\ \|t_{\mathrm{GT}}\left(t_{n}\right)-Qt_{\mathrm{est}}\left(t_{n}\right)+\mu \| \end{array}\right),$$
  where Q and μ are the trajectory alignment parameters recovered from the Procrustes alignment step.
- Relative Pose Error (RPE): The ATE is not very robust as a large number of absolute locations may easily be affected by only few gross estimation errors along the trajectory. The RPE in turn evaluates relative pose estimates between pairs of frames in the sequence. Particularly if the chosen pairs of frames are drawn from local windows, the RPE is a good measure of average local tracking accuracy:
  (14) $${\mathrm{RPE}}_{t}=\left\{\begin{array}{c} \mathrm{mean}\\ \mathrm{median}\\ \mathrm{std}-\mathrm{dev} \end{array}\right\}\left(\begin{array}{c} \|R_{GT}^{T}\left[0\right]\left(t_{GT}\left[k\right]-t_{GT}\left[0\right]\right)\\ -R_{est}^{T}\left[0\right]\left(t_{est}\left[k\right]-t_{est}\left[0\right]\right)\|\\ \cdots \\ \|R_{GT}^{T}\left[n-k\right]\left(t_{GT}\left[n\right]-t_{GT}\left[n-k\right]\right)\\ -R_{est}^{T}\left[n-k\right]\left(t_{est}\left[n\right]-t_{est}\left[n-k\right]\right)\| \end{array}\right)$$
  $${\mathrm{RPE}}_{R}=\left\{\begin{array}{c} \mathrm{mean}\\ \mathrm{median}\\ \mathrm{std}-\mathrm{dev} \end{array}\right\}\left(\begin{array}{c} \left\|\log(R_{GT}^{T}\left[0\right]R_{GT}\left[k\right]{\left(R_{est}^{T}\left[0\right]R_{est}\left[k\right]\right)}^{T})\right\|\\ \cdots \\ \|\log(R_{GT}^{T}\left[n-k\right]R_{GT}\left[n\right]{\left(R_{est}^{T}\left[n-k\right]R_{est}\left[n\right]\right)}^{T})\|, \end{array}\right)$$
  where log(·) represents the Riemannian logarithmic map.

#### 4.4.2. Scene Label Distribution

**Figure 13:**
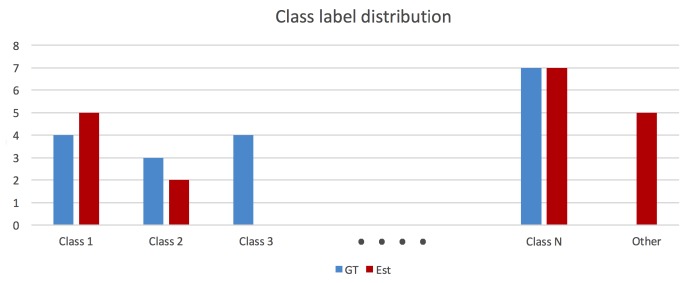
Abstract ground truth and estimated class label distributions.

In order to evaluate the overall capacity of a Spatial AI algorithm to parse a scene and gain a correct understanding of the composition and semantics of the individual objects, we propose to take the Intersection over Union (IoU) between the ground truth and the inferred label distributions. This measure identifies whether or not certain object classes have been identified, whether all of them have been identified, or whether some of them have a nonunique representation. Note that all class labels that are not present in the ground truth set of labels are simply being replaced by the label “other”. In addition to the overall IoU for the entire label distribution, the analysis is completed by individual IoUs for each class. An example distribution is indicated in Figure 13. If $c_{i}$ and $c_{i}^{\prime }$ denote the ground truth and estimated class label counts, the per-class IoU is given by $IoU_{class\;i}=\frac{min(c_{i},c_{i}^{\prime })}{max(c_{i},c_{i}^{\prime })}$, and the overall semantic labelling capability is given by $IoU=\frac{\sum_{i}min\left(c_{i},c_{i}^{\prime }\right)}{\sum_{i}max\left(c_{i},c_{i}^{\prime }\right)}$.

#### 4.4.3. Object Class, Pose, Shape, and Completeness

The most important part of a modern evaluation of a Spatial AI system consists of assessing the quality of each individual object inferred in the scene. However, before any of the inferred objects can be evaluated, we first need to establish correspondences between each inferred object and the best possible correspondence within the ground truth set of objects. These correspondences are established as follows:

- For each object, we pick the nearest and the second nearest object judged by their object centers.
- We first ensure that the distance to the best is below a certain threshold.
- We then ensure that the ratio between the distance to the second best and the distance to the best is below a certain threshold.

For each identified correspondence, we then evaluate the class, pose, shape, and completeness. The object class is trivial to evaluate as it is simply right or wrong. However, other criteria are more expensive to evaluate and require prior steps to transform and align the representation of the shapes. This is due to the fact that the shape and the pose of an object are discerned by the introduction of an object-specific reference frame, and the location and orientation of this reference frame may vary depending on the employed object representation (e.g., some CAD models may be registered in a frame where the *z*-axis points upward, others where it points forward). More importantly, there may be a difference between the convention for this reference frame between the ground truth and the estimated shape representations.

Our shape evaluation procedure starts by transforming the representations into a common reference frame, making their representations comparable, and performing an alignment. The alignment is necessary as the magnitude of the aligning transformation can be used to express the object pose error, and the pose error should be compensated for before we are able to evaluate the shape error. The detailed steps are as follows:

- First, we need to express the geometries in a common reference frame. We reuse the similarity transformation given by Q and μ identified by the prealignment step in the trajectory’s ATE evaluation and combine it with the individual object-to-world transformations to transfer all shape representations into a common global frame.
- The next step consists of expressing geometries in comparable representations. We choose point-clouds as there exist many algorithms and techniques to deal with point clouds. For the ground truth data, we generate point-clouds by simply using the vertices of each CAD mesh. However, in order to ensure sufficiently dense surface sampling, larger polygons are recursively subdivided into smaller subtriangles until the surface of each triangle is below a certain threshold. For the reconstructed model, we also transfer the employed representation into a point cloud:
  – If it is a point cloud already, we leave it unchanged.
  – For a polygon mesh, we simply employ the above-outlined strategy to ensure a sufficiently dense sampling of the surface.
  – For binary occupancy grids, we employ the marching cubes algorithm to transfer the representation into a mesh, and then employ the above-outlined technique to obtain a sufficiently dense point cloud.
  – For a signed distance field, we employ the Poisson surface interpolation method, by which we again obtain a mesh that is transformed as above.
- Once both the ground truth and the estimated object of each correspondence are expressed as point clouds in the same global reference frame, we proceed to their alignment. We choose the GoICP algorithm [73] for their alignment as it enables us to find the globally optimal alignment requiring neither a prior about the transformation nor correspondences between the two-point sets.

Having completed the alignment of each object’s representation, we may finally complete the evaluation of all other criteria:

- Pose: The accuracy of the object pose is simply evaluated by the magnitude of the translation and the angle of rotation of the aligning transformation identified by GoICP [73].
- Shape: The accuracy of the shape is assessed by first applying the aligning transformation identified by GoICP and then taking the mean and median of the Euclidean distances between each point in the estimated point cloud and their nearest neighbour in the ground truth point set. Further shape accuracy measured such as the standard deviation and the maximum and minimum of the point-to-point distances are given as well.
- Completeness: We conclude by assessing the model completeness of each object. This is done by—again—first applying the aligning transformation to the estimated point set. We then mark each point in the ground truth point set as either observed or not. A point is observed if it is within a certain threshold distance of one of the inferred points. The final completeness measure is simply given as
  (15) $$\mathrm{completeness}=\frac{\#\mathrm{observed}\;\mathrm{GT}\;\mathrm{points}}{\#\mathrm{GT}\;\mathrm{points}}$$
  However, this measure obviously depends on a good choice of the threshold distance, which is why the completeness measure is evaluated for multiple such distances. This leads to a curve that expresses the quality of the shape as a function of this radius. The final measure to evaluate the shape completeness or quality is given by taking the area under this curve.

**Figure 14:**
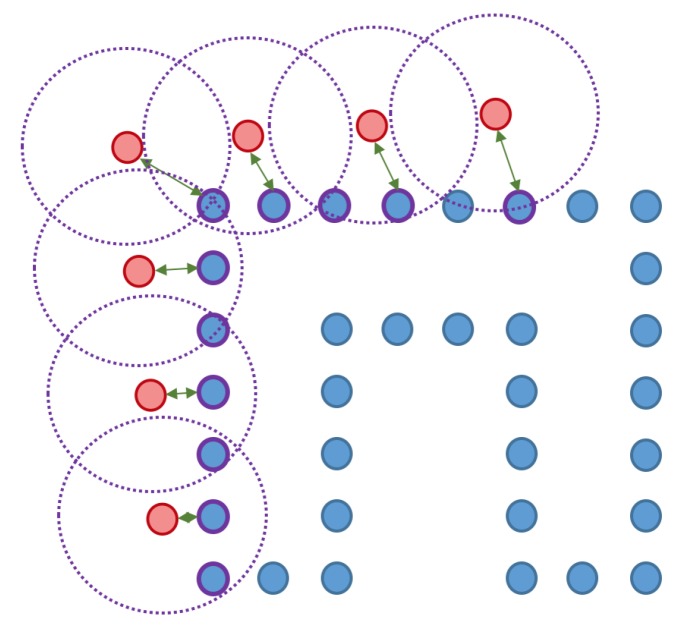
Toy example of the proposed shape accuracy and shape completeness measures. Assume that the estimated (red) and ground truth (blue) points are realigned, the point-to-point distances are given by—for each point in the possibly lower-dimensional estimated point cloud—finding the distance to the nearest neighbour in the ground truth point cloud. Completeness is estimated by marking observed points in the ground truth point cloud as observed if they are within a certain radius of an estimated point (illustrated in purple).

A toy example for the point-to-point distances as well as the ground truth point marking is illustrated in Figure 14.

#### 4.4.4. Background Model

The final geometric element to assess is the structure of the background. We again start by applying Q and μ to express the background geometry in the ground truth frame. We then remove all objects from the estimated geometry and transform the remaining representation into a point cloud by following the same strategy as for the object point clouds. To conclude, we simply take each 3D point in the estimated point cloud and find its nearest neighbour in the ground truth background model. The quality of the background geometry estimation is finally expressed by taking the mean or median of all distances between nearest-neighbour pairs.

#### 4.4.5. Computational Aspects

As introduced in [1], one of the main requirements of a Spatial AI system is given by computational efficiency. The algorithm ultimately needs to be able to run and infer even complex joint semantic-geometric representations on embedded systems. A complete report of the performance of a Spatial AI system is therefore concluded by indicating the time and memory consumption of the computation.

## 5. Application to An Example Spatial AI System

We conclude our exposition by applying our proposed benchmark to two available, state-of-the-art Spatial AI systems. The first one is given by the Kimera pipeline [37], which performs state-of-the-art tracking followed by an estimation of surfaces in the form of a triangular mesh and a semantic labelling of the individual surface elements. The framework does not maintain an object-level representation nor uses semantic knowledge to select tailored representations for individual objects. The second framework is given by Deep-SLAM++ [51], which first infers the object-level composition of the scene and then represents each model by a class-specific low-dimensional latent representation. While Kimera is a bottom-up framework aiming at high accuracy, Deep-SLAM++ is better at inferring scene compositions and complete geometries. The frameworks are evaluated over the baseline experiment given by dataset rm4.

### 5.1. Application to Kimera

**Figure 15:**
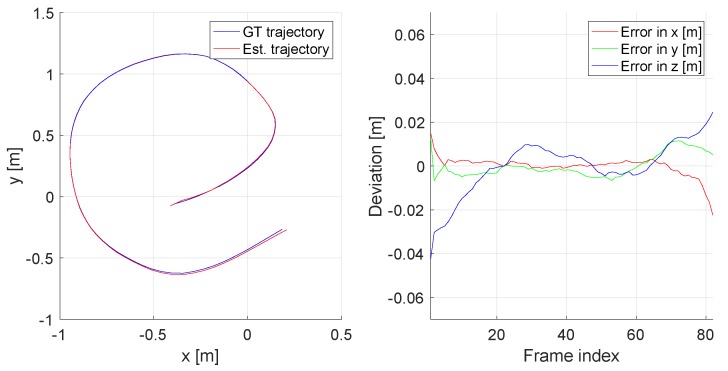
Estimated (red) and ground-truth (blue) trajectories (left), as well as errors over time (right). Dataset: rm4. Unit: [m].

The Kimera pipeline includes a high-performance tracking module that is able to work in either monocular, monocular-inertial, stereo, stereo-inertial, or RGBD modes. We use the stereo-inertial mode as it the most stable one. Kimera achieves highly accurate tracking for which even the ATE measures indicate very low drift errors. The ground truth and the estimated trajectories, as well as their deviations along the trajectory, are illustrated in Figure 15 and summarised in Table 4.

While Kimera is able to estimate highly accurate trajectories, the geometry representation remains primarily a geometrical one. The pipeline relies on a module denoted the mesher to extract dense surface geometries and furthermore assigns a semantic class for each polygon from the originally image-based semantic annotations. However, the framework does not employ any prior shape knowledge, which therefore leads to generally incomplete and inferior object geometries. We therefore complement the Kimera tracker and the image-based semantic labels by an additional module taken from the Deep-SLAM++ pipeline [51].

### 5.2. Application to Deep-SLAM++

In order to overcome the weakness of purely geometric pipelines such as SemanticFusion [33], Fusion++ [34], and Kimera [37] and incorporate the benefits of imposing higher-order knowledge about the geometry of classes of objects, we append an additional module from the Deep-SLAM++ pipeline [51] in which the environment representation is changed into an object-level representation, and object shapes are represented by deeply trained shape auto-encoders. The overall steps of this pipeline look as follows:

- We run an object detector over the left images of each estimated stereo frame and use both depth and semantic information in the bounding box to perform a segmentation of each observed object.
- For each detected object, we find all neighbouring frames in which the object is observable, detect redundancies, and initialise unique object representations with a list of frames in which each object is observable.
- We use Pix3D [60] to predict complete object geometries from the partial, image-based observations. For each object, the initialisation is performed by using the centre observing frame.
- We refine the latent code of each object shape representation by minimising depth errors in all observing frames. This finds a compromise between measurement fidelity and the application of prior shape knowledge.
- To conclude, the individual object shape optimisations are alternated by object pose optimisations in which the entire object shape is aligned with their respective observations.

For further details on the approach, the reader is kindly referred to the work of Lan et al. [51].

**Figure 16:**
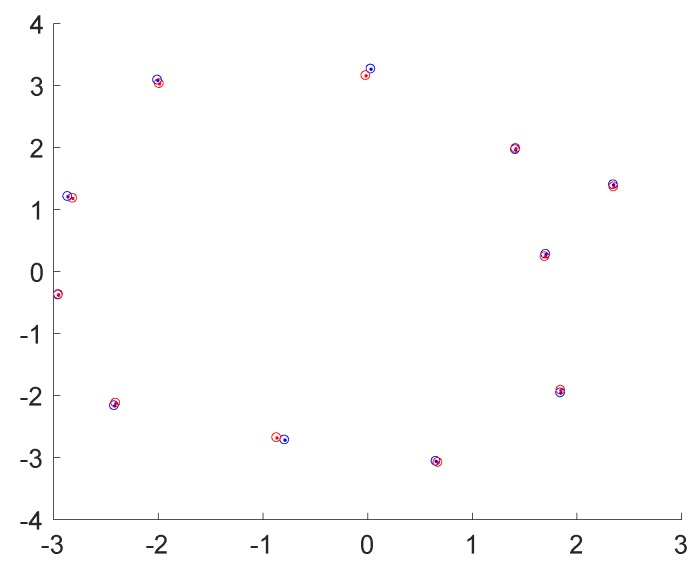
Birdeye view onto the environment with estimated (red) and groundtruth (blue) locations of the objects using the approach of Lan et al. [51]. Dataset: rm4. Unit: [m].

**Figure 17:**
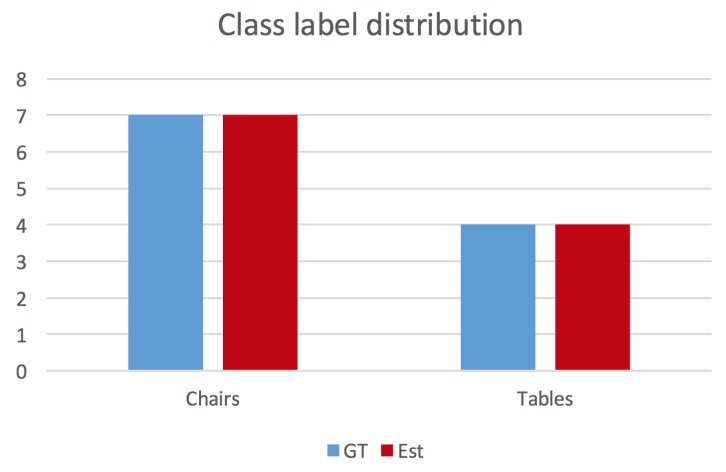
Ground truth and inferred class label distributions for dataset rm4 using the approach of Lan et al. [51].

Figure 16 illustrates a map of the ground truth and the detected object centres, and Figure 17 indicates the ground truth and estimated label distributions. As can be observed, the framework has no difficulties with the baseline dataset rm4, in which no object occlusions are happening. The framework identifies the correct label distribution, and all objects are estimated at approximately correct locations.

**Figure 18:**
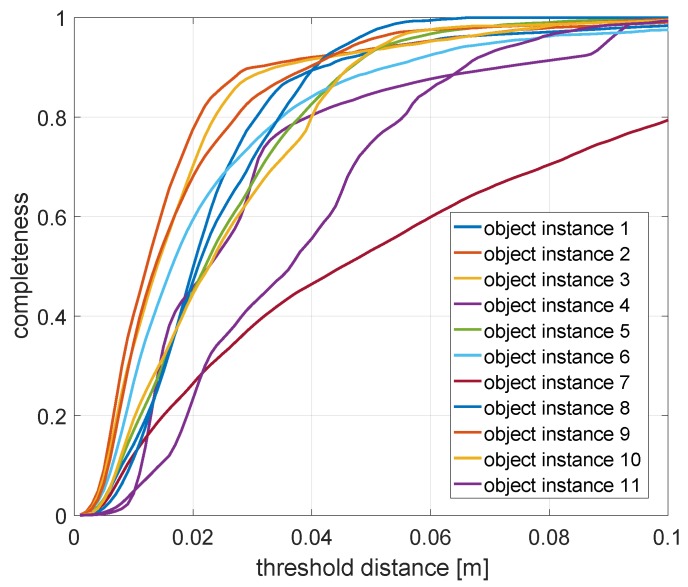
Detailed shape completeness measures as a function of the threshold distance. The area-under-curve serves as a final measure to evaluate the completeness and quality of all 11 object instances.

The most interesting part of the evaluation is finally given by evaluating the accuracy of the object poses and shapes. Note that the accuracy of the object poses is additionally influenced by the slight drift in the camera pose estimations, and it is hard to discern this influence from actual object pose inaccuracies. The results are summarised in Table 5. As can be observed, each object class is correctly identified, and reasonable shapes are estimated as long as the object geometry is sufficiently represented in the training set for the employed Pix3D network. The detailed shape completeness measures as a function of the threshold distance for the area-under-curve computation are illustrated in Figure 18. An exception is given by object instance 7, which is a round table. The network has been trained only on square tables and is hence unable to represent this shape. Overall, the framework achieves relatively low pose errors while $d_{max}$ and the shape completeness measures suggest that there is still space for improvement on the quality of the object shapes. This is primarily due to the limited output resolution of the Pix3D network as well as a generally degrading performance on realistic datasets that contain a domain gap with respect to the training examples.

## 6. Discussion

Current Spatial AI systems vary strongly in their approach. While some frameworks start from more traditional SLAM solutions and infer semantics and object-level partitioning in a bottom-up scheme, others aim at an immediate understanding of the topological, object-level composition of the environment thus enabling the representation of each individual object using a tailored, class-specific representation of each object’s pose and shape. The strong variability of these approaches contrasts with a lack of benchmark datasets for which all information such as semantic class distributions, object-level composition, as well as object shapes are known. We have introduced realistic novel synthetic indoor datasets for which such information is readily available. Combined with the proposed evaluation criteria, we introduce a complete framework for benchmarking Spatial AI systems assessing semantic, geometric, and topological aspects of the estimation and complete our toolchain by flexible scripts to create novel datasets.

## 7. Conclusions

We have combined state-of-the-art solutions and techniques from the communities of visual-inertial tracking, deep learning, and semantic SLAM to achieve a first genuine Spatial AI result in which scenes are represented as a composition of objects with poses and shapes. Our conclusion is that there is still substantial research to be done until both the quality and the computational efficiency of such frameworks will meet the demands of most real-world applications, and it is our hope that this work will be helpful in comparing and driving the future development of Spatial AI systems.

## Figures and Tables

**Table 1:** Provided RGB-DS Datasets.

Datasets	Duration [s]	Semantic Objects	Properties
Single Room Datasets			
**rm1**	70	2C1, 1C2, 1T1, 1T2, 1S1, 1TV1	6.4 × 6.5 × 3.1 m slight shaking slight occlusion
**rm2**	60	1C1, 2C2, 2C3, 1C4, 1T1, 1T2	8.0 × 6.0 × 3.2 m heavy shaking slight occlusion
**rm3**	40	8C1, 2C2, 1C3, 1T1, 1T2, 4T3 2T4, 1T5, 4T6, 1S1	10.0 × 14.0 × 2.8 m slight shaking heavy occlusion
**rm4**	16.7	3C1, 2C2, 2C3, 2T1, 1T2, 1T3	12.1 × 12.0 × 3.2 m no shaking no occlusion
Multiroom Dataset			
**rm5**	60	2C1, 1C2, 2C3, 1C4, 2C5, 1C6 1T1, 1T2, 1T3, 1T4, 1S1, 1TV1	combo of rm1 and rm2 slight shaking slight occlusion

In the column “Semantic Objects”, C = Chair, T = Table, S = Sofa, TV = TV. e.g., 2C1 = two chairs of type “1”. In the column “Properties”, the first set of parameters (e.g., 6.4×6.5×3.1 m refers the volume of the room); “shakiness” refers to the jerkiness of the camera motion (no shaking=camera on the gimbal, heavy shaking=hand-held motion); “occlusions” refer to the semantic objects’ occlusions in the camera views and the overall degree of clutter in the scene.

**Table 2:** CRF test in different datasets.

	SSIM	MSE	PSNR	SSIM	MSE	PSNR
**rm1**	0.9514	71.3171	29.5989	0.9530	23.5196	34.4165
**rm2**	0.8980	720.4544	19.5547	0.8990	625.2354	20.1704
**rm3**	0.9258	436.6094	21.7299	0.8677	167.2929	19.2812
**rm4**	0.9031	327.233	22.4227	0.9593	177.5349	25.6380
**rm5**	0.9191	230.0157	24.5132	0.9264	35.2029	32.6650

The similarity of CRF recovered raw image and Blender generated raw image, SSIM is structural similarity index, MSE is the mean-squared error, PSNR is the peak signal-to-noise ratio, the left three columns are errors of facing sunlight frames, the right three columns are errors of backing sunlight frames.

**Table 3:** Average durations for single image rendering.

	RGB	Depth	Seg
**rm1**	5.07	4.75	2.31
**rm2**	5.07	1.79	1.15
**rm3**	10.17	8.89	4.72
**rm4**	7.87	7.19	1.29
**rm5**	6.57	6.54	2.89
**RGB noise**	5.02	**Depth noise**	16.32

**Table 4:** Absolute Trajectory Errors (ATE) for Kimera. Dataset: rm4. Unit: [m].

Parameter	Value	Parameter	Value
rmse	0.014061	std.-dev.	0.0088741
mean error	0.010951	min error	0.00018726
median error	0.0071863	max error	0.046873

**Table 5:** Analysis of the objects semantically reconstructed on dataset rm4.

Instance	GT Model	Estimated Model	Alignment	Errors & Completeness
1 GT: chair1 Est: chair	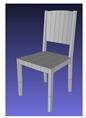	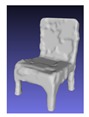	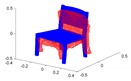	$$\begin{array}{ccc} t_{err} & = & 0.041895,R_{err}=0.096319\\ d_{mean} & = & 0.025936,d_{med}=0.0222\\ d_{std} & = & 0.015678,d_{max}=0.095333\\ d_{min} & = & 0.00032615,\mathrm{compl}=0.7659 \end{array}$$
2 GT: chair1 Est: chair	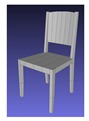	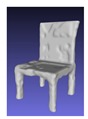	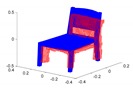	$$\begin{array}{ccc} t_{err} & = & 0.060021,R_{err}=0.056609\\ d_{mean} & = & 0.015612,d_{med}=0.012115\\ d_{std} & = & 0.012943,d_{max}=0.064623\\ d_{min} & = & 0.00032818,\mathrm{compl}=0.8340 \end{array}$$
3 GT: chair1 Est: chair	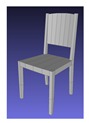	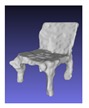	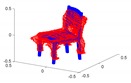	$$\begin{array}{ccc} t_{err} & = & 0.041323,R_{err}=0.052128\\ d_{mean} & = & 0.023013,d_{med}=0.018508\\ d_{std} & = & 0.017844,d_{max}=0.11128\\ d_{min} & = & 0.0004208,\mathrm{compl}=0.8203 \end{array}$$
4 GT: table1 Est: table	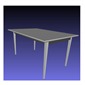	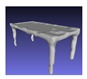	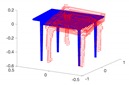	$$\begin{array}{ccc} t_{err} & = & 0.13587,R_{err}=0.10133\\ d_{mean} & = & 0.024858,d_{med}=0.012205\\ d_{std} & = & 0.021418,d_{max}=0.10633\\ d_{min} & = & 0.00064865,\mathrm{compl}=0.7059 \end{array}$$
5 GT: chair2 Est: chair	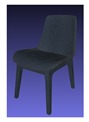	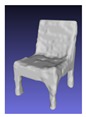	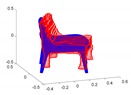	$$\begin{array}{ccc} t_{err} & = & 0.071636,R_{err}=0.20242\\ d_{mean} & = & 0.025514,d_{med}=0.022047\\ d_{std} & = & 0.017295,d_{max}=0.089588\\ d_{min} & = & 0.00028348,\mathrm{compl}=0.7519 \end{array}$$
6 GT: chair3 Est: chair	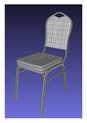	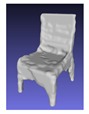	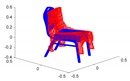	$$\begin{array}{ccc} t_{err} & = & 0.046181,R_{err}=0.12623\\ d_{mean} & = & 0.025655,d_{med}=0.022377\\ d_{std} & = & 0.017597,d_{max}=0.10529\\ d_{min} & = & 0.00035565,\mathrm{compl}=0.7665 \end{array}$$
7 GT: table3 Est: table	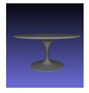	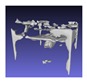	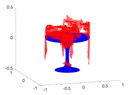	$$\begin{array}{ccc} t_{err} & = & 0.12321,R_{err}=0.055047\\ d_{mean} & = & 0.037219,d_{med}=0.030943\\ d_{std} & = & 0.030366,d_{max}=0.18215\\ d_{min} & = & 0,\mathrm{compl}=0.4905 \end{array}$$
8 GT: table1 Est: table	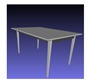	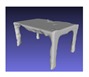	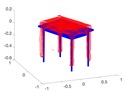	$$\begin{array}{ccc} t_{err} & = & 0.070875,R_{err}=0.031555\\ d_{mean} & = & 0.023082,d_{med}=0.01876\\ d_{std} & = & 0.016171,d_{max}=0.10508\\ d_{min} & = & 0,\mathrm{compl}=0.7708 \end{array}$$
9 GT: chair2 Est: chair	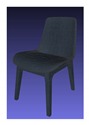	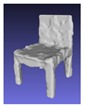	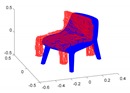	$$\begin{array}{ccc} t_{err} & = & 0.097072,R_{err}=0.098674\\ d_{mean} & = & 0.01585,d_{med}=0.011098\\ d_{std} & = & 0.014224,d_{max}=0.10307\\ d_{min} & = & 0.00023002,\mathrm{compl}=0.8179 \end{array}$$
10 GT: chair3 Est: chair	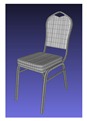	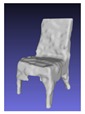	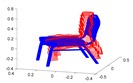	$$\begin{array}{ccc} t_{err} & = & 0.052934,R_{err}=0.47158\\ d_{mean} & = & 0.038789,d_{med}=0.036827\\ d_{std} & = & 0.023687,d_{max}=0.18855\\ d_{min} & = & 0,\mathrm{compl}=0.7478 \end{array}$$
11 GT: table2 Est: table	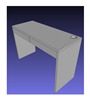	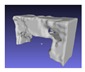	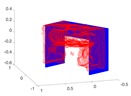	$$\begin{array}{ccc} t_{err} & = & 0.098702,R_{err}=0.075488\\ d_{mean} & = & 0.044182,d_{med}=0.034869\\ d_{std} & = & 0.037135,d_{max}=0.21209\\ d_{min} & = & 0,\mathrm{compl}=0.6275 \end{array}$$
